# Stretched penile length and total serum testosterone in term male neonates

**DOI:** 10.11604/pamj.2020.37.61.21123

**Published:** 2020-09-15

**Authors:** Abiodun John Kareem, Jerome Boluwaji Elutayo Elusiyan, Adesola Olawumi Kareem

**Affiliations:** 1 Endocrinology and Metabolism Unit, Department of Paediatrics, Obafemi Awolowo University Teaching Hospitals Complex, Ile-Ife, Osun State, Nigeria,; 2 Department of Paediatrics and Child Health, Obafemi Awolowo University, Ile-Ife, Osun State, Nigeria,; 3 Department of Community Health, Federal Medical Centre, Owo, Ondo-State, Nigeria

**Keywords:** Term male neonates, total serum testosterone, stretched penile length

## Abstract

**Introduction:**

the reference values of stretched penile length vary with different ethnic group. There is paucity of data on the reference range of total serum testosterone in neonates especially in Africa. This study therefore was to determine the normal stretched penile length, total serum testosterone levels in term male newborns and to correlate them with anthropometric parameters.

**Methods:**

this was a prospective cross-sectional study. One hundred and twenty-four consecutive healthy term male neonates were recruited in the first 72 hours of postnatal life. The stretched penile length (SPL) was measured with a rigid metric ruler. Weight, length and occipitofrontal circumference were also measured. Total serum testosterone level was determined using Enzyme Linked Immunoassay. Data were analysed using the Statistical Package for Social Sciences for Windows version 20.

**Results:**

a total of 124 term male neonates were recruited. The postnatal age of recruited neonates was one to 70 hours with a mean of 22.8 ± 17.6 hours and the mean of estimated gestation age was 38.5 ± 1.3 weeks. The range of stretched penile length was from 2.1 to 3.9 cm with a mean of 3.2 ± 0.4 cm and SPL less than 2.2 cm was considered as micropenis. The mean total serum testosterone level was 357.4 ± 241.7 ng/dl. The SPL had a positive correlation with the birth weight, length and total serum testosterone. The total serum testosterone and birth length were predictors of stretched penile length.

**Conclusion:**

among the studied population the mean stretched penile length was 3.2 cm and mean total serum testosterone was 357.4 ng/dL.

## Introduction

Evaluation of the external genitalia is a pertinent component of the newborn examination [1]. The exact penile size is an important measurement for diagnosing genital problems such as micropenis and ambiguous genitalia [2]. Micropenis is defined as stretched penile length (SPL) of at least 2.5 standard deviation below the mean for age, with normal structure and function or SPL less than third percentile for SPL [3,4]. Micropenis might be the only obvious feature of hypothalamus-pituitary axis disorder with multiple pituitary hormone disorder [5]. Abnormal penile size in neonate may be a sign of serious systemic problem with dire medical, social and psychological consequences [6,7]. The development of normal penis is dependent on testosterone action and testosterone receptor [8]. Insufficient testosterone action during the second and third trimesters in the male foetus leads to undervirilisation at birth and disorders of sexual differentiation such as hypospadias and small penis [2,9].

It is important to note that normal penile length may vary with different populations and races [2]. Studies from Korea [10], Turkey [11] and among the Igbos in Nigeria [12] showed different normal values of the SPL but the testosterone levels were not analysed in the studies [10-12]. The different normal SPL represent the population studied. There are few reference data on the range of penile lengths [2,4,12] but no available data about the reference range of total serum testosterone in term Nigerian neonates. The available reference ranges of total serum testosterone in neonates are Caucasian values. Hence, this study aimed to determine the reference values for total serum testosterone in term male Nigerian neonates, the SPL and their correlation with the anthropometric parameters.

## Methods

This was a cross-sectional prospective study carried out at the labour and postnatal wards of the Ife Hospital Unit (IHU) of the Obafemi Awolowo University Teaching Hospitals Complex (OAUTHC), Ile-Ife, Nigeria between July 1^st^, 2017 and October 31^st^, 2017. Ethical approval with registration number IRB/IEC/0004553 was obtained from the Hospital´s Ethics Committee and written informed consent was obtained from the parents of each baby that was recruited for the study. One hundred and twenty-four consecutive apparently healthy male neonates delivered at term between 37 and 42 completed weeks of gestation were recruited within the first 72 hours of postnatal life. Neonates of mothers who received or were receiving androgenic medication such as Fluoxymesterone (androxy), androgel or depotestogen during pregnancy and neonates with dysmorphic features or anomalies of external genitalia such as hypospadias, undermasculinisation of the external genitalia and cryptorchidism were excluded. A detailed history and a thorough physical examination especially examination of the genitalia including position of the urethral opening, skin of the scrotum, location of the testes and number of the testes was carried out for each neonate. The SPL, birth weight, birth length and occipitofrontal circumference (OFC) were also determined for each neonate. All the measurements were done according to standard methods as described below. The SPL was measured with a rigid metric ruler by two different research doctors who were trained within the department. The research doctors measured the SPL of the neonates separately and the mean value of the two measurements of each SPL was used. The SPL was measured by using one end of the ruler to maximally depress the pubic pad of fat via the pubic ramus at the base of the penis, the penis was fully stretched to the point of increased resistance and the distance to the tip of the glans of the stretched penis on the dorsal surface was plotted on the other end of the ruler [13]. The foreskin of the penis was not included in the measurement. The measurement was done with the neonate in supine position and movement restrained with the help of an assistant.

Three millilitres of blood sample was collected from each neonate between 0530 and 0800 hours to allow for consistency within the circadian rhythm. The serum was separated and kept at -20^ο^C until the time of analysis at the Department of Chemical Pathology of OAUTHC, Ile-Ife. Total serum testosterone level was measured using Enzyme Linked Immunoassay (ELISA) system according to recommendation of the manufacturer of the kit (ACCU-BIND ELISA Microwells Testosterone Test System kits with product code 3725-300 manufactured by Monobind Inc. in Lake Forest, CA 92630, USA). Analytical accuracy was ensured by running controls (normal and high) obtained from Randox Laboratory Limited, UK along with the assay. The control values were within acceptable range written in the kit manual before accepting the assay result. Precision of the method was determined using controls with the interassay Coefficient of Variation (COV) being 3.2%. Data were analysed using the Statistical Package for Social Sciences (SPSS) for Windows version 20. Mean and standard deviation (SD) were determined for continuous variables like postnatal age, estimated gestational age, birth weight, birth length, occipitofrontal circumference (OFC), stretched penile length and total serum testosterone levels. The percentile of the stretched penile length was determined. The relationship between stretched penile length and birth weight group was determined using one-way Anova. The relationship between stretched penile length, birth weight, birth length and total serum testosterone levels were determined using Pearson´s correlation coefficient (r). Multiple linear regression analysis was used to determine predictors of SPL. Scatter plots of stretched penile length against total serum testosterone levels, birth weight and length of the neonates were done. Probability (p) value less than 0.05 was accepted as statistically significant.

## Results

**Figure 1:**
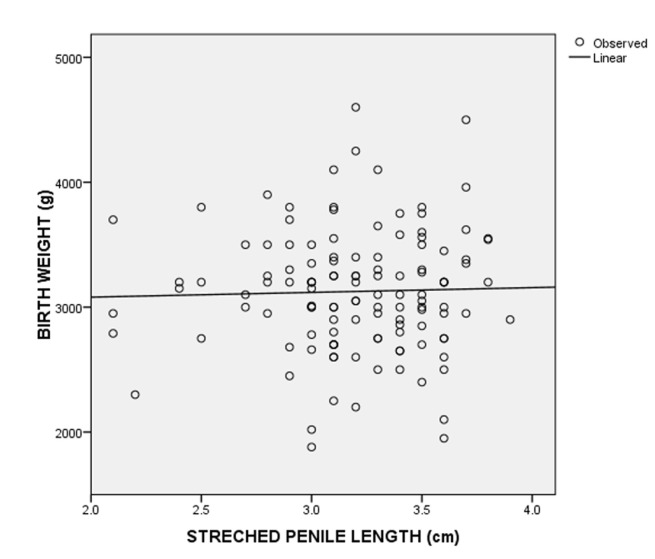
scatter plots of birth weight against stretched penile length and the best-fitted regression line in neonates

**Figure 2:**
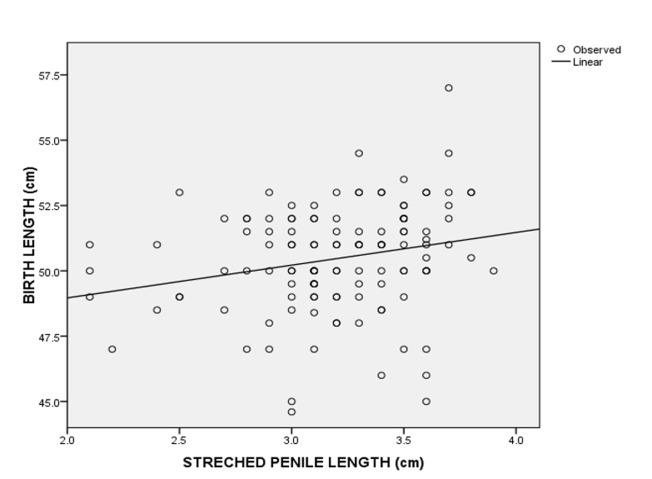
scatter plots of birth length against stretched penile length and the best-fitted regression line in neonates

**Figure 3:**
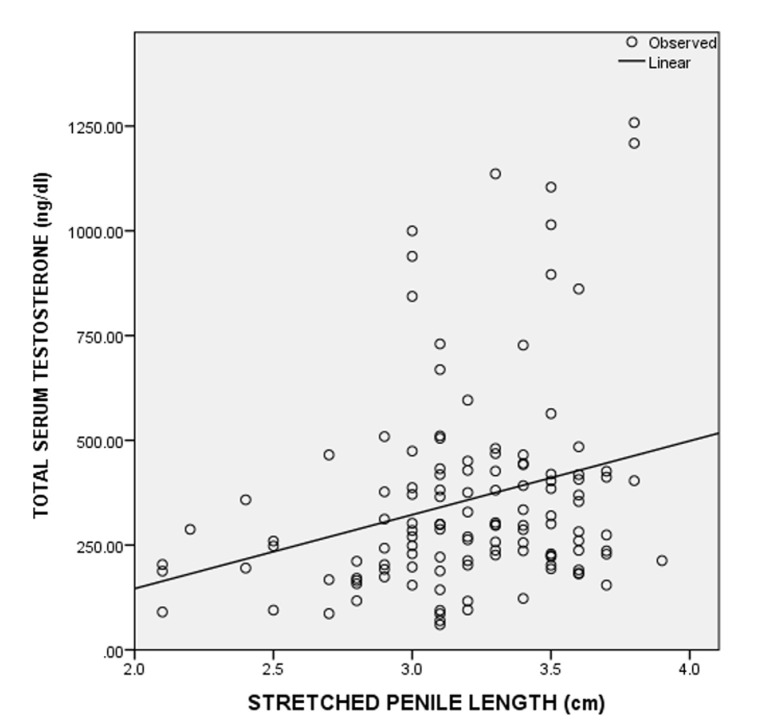
scatter plots of total serum testosterone concentrations against stretched penile length and the best-fitted regression line in neonates

A total of 124 consecutive male neonates were recruited for the study. The postnatal age of the neonates ranged from 1 to 70 hours. Eighty-three (66.9%) of the neonates were aged less than 24 hours, 28 (22.6%) were aged 24 to 47 hours and 13 (10.5%) were from 48 to 70 hours. The mean postnatal age of the neonates was 22.8 ± 17.6 hours. The demographic characteristics of the neonates are shown in Table 1. The estimated gestation age ranged from 37 to 42 weeks of which sixty-nine (55.6%) of the neonates had gestational age ≤ 38 weeks, while 55 (44.4%) had gestational age > 38 weeks. The mean of SPL was 3.2 ± 0.4 cm. A SPL less than 2.2 cm was at least 2.5 SD below the mean SPL and was considered as micropenis. The third, 50^th^ and 97^th^ percentiles of the SPL were 2.2 cm, 3.2 cm and 3.8 cm, respectively. The birth weight of the neonates ranged from 1.9 to 4.6 kg and about 110 (90%) of the neonates had birth weight between 2.5 and 4.0 kg (Table 2). There was no statistically significant difference in the SPL across the various birth weight groups (Table 3). The SPL had a weak positive correlation with the birth weight (r=0.029, p=0.750) (Figure 1) and birth length (r=0.227, p=0.011) (Figure 2). The SPL correlated positively with the total serum testosterone levels though weak (r=0.271, p=0.002) (Figure 3). Table 4 shows the multiple regression analysis of stretched penile length, birth length and total serum testosterone of the neonates. A multilinear regression analysis was conducted to predict SPL based on the birth length and total serum testosterone. A significant regression equation was found [F (2,121) = 8.671, < 0.001'> P < 0.001] with a R^2^ = 0.125. Participants´ predicted SPL was equal to 0.966 + 0.041 (birth length) + 0.001 (total serum testosterone) where birth length was measured in centimetres and total serum testosterone in ng/dL. The participants´ SPL increased 0.041cm for each centimetres of birth length and 0.001cm for each ng/dL of total serum testosterone. Birth length and serum testosterone were significant predictors of SPL.

**Table 1:** descriptive statistics of the study group

Variables	Mean±SD	Range
Gestational age (weeks)	38.3±1.2	37.0-42.0
Maternal age (years)	30.4±4.9	19.0-42.0
Birth weight (kg)	3.1±0.5	1.9-4.6
Birth length (cm)	50.5±2.1	44.6-57.0
Head circumference (cm)	34.6±1.3	31.0-37.9
SPL (cm)	3.2±0.4	2.1-3.9
Total serum testosterone (ng/dL)	357.4±241.7	59.5-1258.3

**Table 2:** birth weight of the neonates

Birth weight group (g)	Frequency (%)
<2500	9(7.3)
2500-4000	110 (88.7)
>4000	5 (4.0)
Total	124 (100.0)

**Table 3:** relationship between stretched penile length and birth weight of the neonates

	Sum of Squares	df	Mean Square	F	Sig.
Between Groups	0.058	2	0.029	0.177	0.838
Within Groups	19.882	121	0.164		
**Total**	19.940	123			

df= degree of freedom; F= One-way Anova

**Table 4:** multiple linear regression analysis of stretched penile length, birth length and total serum testosterone of the neonates

Model	Unstandardized Coefficients		Standardized Coefficients	t	Sig.	95.0% Confidence Interval for B	
	B	Std. Error	Beta			Lower Bound	Upper Bound
(Constant)	0.966	0.781		1.236	0.219	-0.581	2.513
Length (cm)	0.041	0.015	0.227	2.673	0.009	0.011	0.072
Total serum testosterone (ng/dl)	0.001	0.001	0.271	3.191	0.002	0.000	0.001

a. Dependent Variable: stretched penile length (cm)

b. Predictors: total serum testosterone (ng/dl), length (cm)

## Discussion

Accurate measurement of the phallic length is important in determining abnormal penile size and in monitoring the management of an underlying disease. Reporting abnormal penile length is a great concern to both the physician and the parents. Therefore, the examiner must report accurate penile length for determining abnormal penile sizes. The mean SPL of the male neonates in our study was 3.20 cm. This was similar to studies from Iran [14], Turkey [1], Egypt [15] and Nigeria [16]. Our result was however lower than values reported from Malaysia [5], Ghana [17], other studies from Nigeria [2,12] and Turkey [11]. The SPL value in our study was higher than studies from Asia like Japan with mean of SPL of 3.06 cm [18], Taiwan with mean of SPL of 3.0 cm [19] and China with mean of SPL of 3.0 cm [20]. The variations in SPL within and between different populations may not be due to the method used in the measurement of the SPL only but could be multifactorial-including environmental, climatic, race and genetics [2,21]. On the other hand, Davarci *et al*. refuted the ethnic, race or environmental differences accounting for the variations of the SPL because of the lack of historical data, rather suggesting that the study population size, selection of participant and methodological variation might account for the SPL variations [22]. From this discussion, a single standard methodology for measurement of the SPL may be challenged. Micropenis is defined as SPL of at least 2.5 standard deviation below the mean for age, with normal structure and function or SPL less than third percentile for SPL [3,4]. From the definition above, our study showed that a male neonate with SPL less than 2.2 cm has micropenis. This value will help in prompt diagnosis and in preventing over-estimation of micropenis which could lead to unnecessary investigations or anxiety.

In our study the mean total serum testosterone concentrations was 357.4 ± 241.7 ng/dL which was higher than total serum testosterone levels from Zaria in Northern Nigeria [23] and Egypt [15]. There was no other published data to the best of our knowledge on the value of total serum testosterone among neonates in Africa. The higher mean of total serum testosterone concentrations in our study when compared to other studies [15,23] could be due to the fact that in our study blood samples were taken at the peak hours of testosterone production whereas other studies [15,23] did not put the circadian rhythm of serum testosterone concentration into consideration. The variation in day of sampling (0-72 hours post-birth) in the present study may also have had an impact on the range of values obtained. The variations in the assay used may also explain differences between studies. The total serum testosterone concentrations in our study ranged from 59.9 to 1258.3 ng/dL while that from Egypt by Maha *et al*. [15] was from 100 ng/dL to 660 ng/dL which were higher than the reference values of total serum testosterone concentrations among the Caucasians males with reference values of 75-400 ng/dL [24]. This could suggest that the serum testosterone concentrations of African are higher than the Caucasians [25]. Our study may be a step for providence of reference range of total serum testosterone concentrations in African populations. Studies from Egypt by Maha *et al*. [15] and Kholy *et al*. [26] showed weak positive correlation between the total serum testosterone concentration and the SPL. These are in concordance with our study, which showed a positive correlation between the total serum testosterone concentration and the SPL though weak. The strength of the correlation which was weak would suggest that other factors other than the total serum testosterone could determine the SPL of the neonates. The multiple linear regression analysis in our study however showed that the total serum testosterone and birth length were predictors of SPL. This would suggest that the penile length is dependent on serum testosterone level, conversion of testosterone by 5-alpha reductase to dihydrotestosterone and a functional androgen receptor [8] and the birth length.

In our study the SPL had a weak positive correlation with birth length and birth weight similar to studies from Singapore [27], Turkey [1], Egypt [15], Ghana [17] and among the Igbo ethnic group in Nigeria [12] but contrary to what Elusiyan *et al*. [4] reported where there was no correlation between SPL and birth length and birth weight. The difference in the correlation could be due to the use of vernier caliper in the measurement of SPL in the study by Elusiyan *et al*. [4] as against rigid metric rule in other studies that could be affected by inter/intra observer variations. However, in our study the bias of the inter/intra observer variation was reduced by ensuring proper training of the research doctors and having two doctors do the measurement and taking average measurement of both researchers. It could also be due to increased subcutaneous fat in the pubic area in interfering with accurate measurement. From the multiple linear regression analysis in our study the birth length was a predictor of SPL. This finding suggested that birth length plays an important role in the SPL of male neonates. The limitation to this study is the small sample size which is used to determine normal values for penile length and total serum testosterone. Further multicentre study is needed to determine the total serum testosterone and stretched penile length among neonates on a larger scale.

## Conclusion

In conclusion the mean stretched penile length among the studied population was 3.2 cm and the mean total serum testosterone was 357.4 ng/dL. A stretched penile length less than 2.2 cm should be taken as representing micropenis. This study is a step-in achieving reference value for stretched penile length and total serum testosterone in term male neonates.

### What is known about this topic

- The average stretched penile length of term male neonates;
- The variation of the stretched penile length with respect to geographical location and ethnic group.

### What this study adds

- The mean stretched penile length of Nigerian term male neonates is 3.2 ± 0.4 cm and SPL less than 2.2 cm could be considered micropenis;
- Total serum testosterone is not the only determinant of stretched penile length of term male neonates;
- The total serum testosterone and birth length are positive determinants of the stretched penile length of term male neonate. The total serum testosterone (ß = 0.271, P = 0.002, 95% Confidence Interval [CI], 0.000 to 0.001) and birth length (ß = 0.227, P = 0.009, 95% CI, 0.01 to 0.07) were significant predictors of the SPL.

## References

[1] Akin Yasemin, Ercan O, Telatar B, Tarhan Faith (2011). Penile size in term newborn infants. Turk J Pediatr.

[2] Jarrett Olatokunbo, Ayoola OO, Jonsson B, Ritzen EM, Albertsson-Wikland Kerstin (2014). Penile size in healthy Nigerian newborns: country-based reference values and international comparisons. Foundation Acta Paediatr.

[3] Tsang Shirley (2010). When size matters: a clinical review of pathological micropenis. J Pediatr Health Care.

[4] Jerome Boluwaji Elutayo Elusiyan, Foluke Grace Ojetayo, Akinwumi Oladapo Fajola (2016). Penile dimension of newborns at Obio Cottage Hospital, Port Harcourt, Nigeria. Niger Postgrad Med J.

[5] Ting Tzer, Wu Loo (2009). Penile length of term newborn infants in multiracial Malaysia. Singapore Med J.

[6] Perovic Sava, Djordjevic Miroslav (2001). Penile lengthening. BJU int.

[7] Shamloul Rany (2005). Treatment of men complaining of short penis. Urol.

[8] Kim Kun, Liu W, Cunha GR, Russell DW, Huang H, Shapiro Ellen (2002). Expression of the androgen receptor and 5 alpha-reductase type 2 in the developing human fetal penis and urethra. Cell Tissue Res.

[9] Keith LM, Persaud TVN, Mark GT (2015). The Developing Human Clinically Oriented Embryology- 10^th^ edition.

[10] Park Jae, Lim G, Oh KW, Ryu DS, Park S, Jeon Jong (2015). Penile length, digit length, and anogenital distance according to birth weight in newborn male infants. Korean J Urol.

[11] Camurdan Aysu, Oz OM, Ilhan MN, Camurdan OM, Sahin F, Beyazova Ufuk (2007). Current stretched penile length: cross-sectional study of 1040 healthy Turkish children aged 0 to 5 years. Urol.

[12] Chikani Ugo, Chinawa JM, Ikefuna AN, Ibekwe Maryann (2015). Stretched penile length of healthy term neonates: normative values among Igbo babies in southeastern Nigeria. J Trop Paediatr.

[13] Analia Tomova, Fnu D, Ralitsa R, Hristina L, Philip K, Ashok Agarwal (2010). Growth and development of male external genitalia: a cross-sectional study of 6200 males aged 0 to 19 years. Arch Pediatr Adolesc Med.

[14] Alaee Ehsan, Gharib MJ, Fouladinejad Mahnaz (2014). Penile length and anogenital distance in male newborns from different Iranian ethnicities in Golestan Province. Iran Red Crescent Med J.

[15] Maha Mohamed, Rania MA, Mohamed TH, Mai Hussein (2015). Penile length and cord total and free testosterone in full term male Egyptian neonates. Egyptian Pediatr Ass.

[16] Ogundoyin Olakayode, Lawal TA, Olulana Dare (2016). Measurement of penile size in healthy Nigerian newborns using conventional penile length measurement technique. Ann Pediatr Surg.

[17] Asafo-Agyei Serwah, Ameyaw E, Chanoine JP, Nguah Samuel (2017). Normative penile anthropometry in term newborns in Kumasi, Ghana: a cross-sectional prospective study. Int J Pediatr Endocrinol.

[18] Nobutake Matsuo, Tomohiro Ishii, John Takayama I, Masayuki Miwa, Tomonobu Hasegawa (2014). Reference standard of penile size and prevalence of buried penis in Japanese newborn male infants. Endocr J.

[19] Chung-Hsing Wang, Wei-De Lin, Da-Tian Bau, Chang-Hai Tsai, Da-Cheng Liu, Fuu-Jen Tsai (2006). Penile length of normal boys in Taiwan. Acta Paediatr Taiwan.

[20] Fok TF, Hon KL, So HK, Wong E, Ng PC, Chang Allan (2005). Normative data of penile length for term Chinese newborns. Biol Neo.

[21] Semiz Serap, Kucuktasc K, Zeneir M, Sevinc Ozgur (2011). One-year follow-up of penis and testis sizes of healthy Turkish male newborns. Turk J Pediatr.

[22] Davarci Mursel, Gokce A, Yalcinkaya FR, Kaya YS, Turhan E, Tutanc Murat (2012). A new anthropometric measurement of penile length and its relation to second and fourth digital lengths. Turk J Med Sci.

[23] Avidime Ohunene, Avidime S, Olorunshola KV, Dikko Au (2011). Anogenital distance and umbilical cord testosterone level in newborns in Zaria, Northern Nigeria. Niger J Physiol Sci.

[24] Esoterix Inc (2018). Expected Value and S I Unit Conversion Table, 5^th^ edition.

[25] Rohrmann Sabine, Sutcliffe CG, Bienstock JL, Monsegue D, Akereyeni F, Bradwin Gary (2009). Racial variation in sex steroid hormones and the insulin-like growth factor axis in umbilical cord blood of male neonates. Cancer Epidemiol Biomarkers Prev.

[26] El Kholy Mohamed, Hamza RT, Saleh M, Elsedfy Heba (2013). Penile length and genital anomalies in Egyptian male newborns: epidemiology and influence of endocrine disruptors. J Pediatr Endocrinol Metab.

[27] Lian Wee, Lee WR, Ho Lai (2000). Penile length of newborns in Singapore. J Pediatr Endocrinol Metab.

